# Whole-Transcriptome Sequencing Reveals the Global Molecular Responses and NAC Transcription Factors Involved in Drought Stress in *Dendrobium catenatum*

**DOI:** 10.3390/antiox13010094

**Published:** 2024-01-12

**Authors:** Siqi Zhang, Yuliang Han, Qinzong Zeng, Chenchang Wang, Huizhong Wang, Juncheng Zhang, Maohong Cai, Jiangjie Lu, Tao Chen

**Affiliations:** Zhejiang Provincial Key Laboratory for Genetic Improvement and Quality Control of Medicinal Plants, College of Life and Environmental Science, Hangzhou Normal University, Hangzhou 311121, China; 2022011010002@stu.hznu.edu.cn (S.Z.); hanyuliang@stu.hznu.edu.cn (Y.H.); zengqz@stu.xju.edu.cn (Q.Z.); 2022111010021@stu.hznu.edu.cn (C.W.); whz20021157@hznu.edu.cn (H.W.); jczhang@hznu.edu.cn (J.Z.); caimaohong@hznu.edu.cn (M.C.)

**Keywords:** *DcNAC*, transcription factor, drought stress, whole transcriptome, *Dendrobium catenatum*

## Abstract

*Dendrobium catenatum* is a highly drought-tolerant herb, which usually grows on cliffs or in the branches of trees, yet the underlying molecular mechanisms for its tolerance remain poorly understood. We conducted a comprehensive study utilizing whole-transcriptome sequencing approaches to investigate the molecular response to extreme drought stress in *D. catenatum*. A large number of differentially expressed mRNAs, lncRNAs, and circRNAs have been identified, and the NAC transcription factor family was highly enriched. Meanwhile, 46 genes were significantly up-regulated in the ABA-activated signaling pathway. In addition to the 89 NAC family members accurately identified in this study, 32 members were found to have different expressions between the CK and extreme drought treatment. They may regulate drought stress through both ABA-dependent and ABA-independent pathways. Moreover, the 32 analyzed differentially expressed *DcNACs* were found to be predominantly expressed in the floral organs and roots. The ceRNA regulatory network showed that *DcNAC87* is at the core of the ceRNA network and is regulated by miR169, miR393, and four lncRNAs. These investigations provided valuable information on the role of NAC transcription factors in *D. catenatum*’s response to drought stress.

## 1. Introduction

*D. catenatum* is a perennial epiphytic herb belonging to the Orchidaceae family, which is classified as a protected plant and has been listed in the Chinese Pharmacopoeia. *D. catenatum* holds significant value as a Chinese herbal medicine, possessing medicinal abilities to lower blood sugar, enhance body immunity, and delay skin aging. *D. catenatum* stands out from other species within the Dendrobium genus due to its exceptional adaptability, robust disease resistance, and vigorous growth vitality. In its natural habitat, *D. catenatum* primarily thrives in challenging environments, such as rocky surfaces, dense bark, tree trunks, or cliff areas [1]. Hence, it is of great significance to study how to overcome the harsh growth conditions of *D. catenatum*, and this may promote our understanding of plant stress tolerance mechanisms.

With the significant impact of global climate change, drought stress has become a critical challenge for plants worldwide. The regulation of plant drought resistance is a complex process involving various substances and pathways that respond to drought stress. This includes the upregulation of polyamines and alterations in the scavenging of reactive oxygen species [2], the high expression of genes related to osmotic metabolism, secondary metabolite synthesis and phytohormone synthesis like that for abscisic acid (ABA), brassinosteroids (BRs), and ethylene [3,4,5]. Plants experiencing drought stress employ multiple defense mechanisms to respond to signals related to drought stress, which are mediated through a series of signal transduction pathways. The rapid expression regulation of mRNA is among the earliest response mechanisms. Apart from the involvement of messenger RNA, noncoding RNA also plays a regulatory role in these processes. DAN1 is a long intergenic noncoding RNA (lincRNA) produced in response to drought stress in allotetraploid upland cotton (*Gossypium hirsutum*) [6]. DRIR, a drought-induced lncRNA, was confirmed as a positive regulator of the plant response to drought stress [7]. Rice lncRNA TCONS_00021861 can modulate plant resistance to drought stress through miR528-3p [8]. The regulatory role of lncRNA in cassava under abiotic stress is discovered by screening RNA expression profiles [9]. lncRNA identified in drought stress by transcriptome data mining has also been reported in Arabidopsis, maize, tomato, *Pyrus betulifolia*, *Oryza nivara*, and foxtail millet (*Setaria italica*) [10,11,12,13,14,15].

The involvement of miRNA in the plant drought response has been extensively investigated in various plant species. For instance, miR159 in tomatoes has been shown to enhance plant drought tolerance by targeting MYB transcription factors [16]. Many potential regulatory miRNAs have also been discovered in multiple species like rice, *triticum dicoccoides*, tomato, Chinese jujube, sugarcane, and *Vicia sativa* [17,18,19,20,21,22]. There are few circRNA studies in plants compared to research reports in animals. circRNAs related to drought stress have been reported in plants such as *Pyrus betulifolia* Bunge, apple, sea buckthorn, *Zea mays*, Alfalfa (*Medicago sativa* L.), and *Phyllostachys aureosulcata* [23,24,25,26,27,28].

Transcriptome analysis is a valuable tool for identifying differentially expressed genes (DEGs) in specific plant tissues and cells. However, there is currently a lack of research on the transcriptome of drought-treated D. catenatum, resulting in limited knowledge regarding the role of different RNA types in the plant’s response to drought stress. Here, we analyzed the whole transcriptome of *D. catenatum* under both well-watered (CK) and drought conditions, aiming to discover the related genes in the transcriptome of *D. catenatum* during drought response regulation. Moreover, we identified the NAC family as the most prominent transcription factor in DEmRNAs, and subsequently their tissue expression patterns in *D. catenatum* and expression patterns under ABA and NaCl treatments were assessed. Our results shed light on the role of the NAC family in drought tolerance and provide valuable insights into the mechanisms underlying RNA-mediated drought tolerance in *D. catenatum* and knowledge for advancing the investigation of genes associated with drought resistance. 

## 2. Methods

### 2.1. Plant Materials

RJ, cultured in bark and sawdust pots at 25 ℃ with a cycle of 16 h/light and 8 h/darkness, was well watered under CK conditions. Then, we conducted extreme drought treatment of two months on several pots cultivated under the same temperature and light conditions for 2 months. 

### 2.2. RNA Extraction, Library Construction, and RNA Sequencing

The total RNA was extracted from *D. catenatum* using RNAprep Pure Plant Plus Kit (Polysaccharides & polyphenolics-rich) (Tiangen, Beijing, China, Cat# DP441) according to the manufacturer’s protocol. RNA purity was checked using the kaiaoK5500^®^ Spectrophotometer (Kaiao, Beijing, China). RNA integrity and concentration were assessed using the RNA Nano 6000 Assay Kit of the Bioanalyzer 2100 system (Agilent, Santa Clara, CA, USA).

For mRNA, lncRNA, and circRNA sequencing, an NEBNext R Ultra^TM^ Directional RNA Library Prep Kit (Illumina, San Diego, CA, USA) for Illumina R (NEB) kit was used to construct a ribosomal RNA removed strand-specific library following the manufacturer’s recommendations. A total of 6 libraries were prepared and sequenced on the Illumina Hiseq 4000 platform with double-ended sequencing read length 2 × 150 bp (PE150). For small RNA sequencing, the Illumina NEBNext Multiplex Small RNA Library Prep Set (NEB) (Illumina, San Diego, CA, USA) was used to prepare six small RNA sequencing libraries following the manufacturer’s recommendations. 

The clustering of the index-coded samples was performed on a cBot cluster generation system using TruSeq PE Cluster Kit v4-cBot-HS (Illumina, San Diego, CA, USA) according to the manufacturer’s instructions. After cluster generation, the libraries were sequenced on an Illumina Hiseq 4000 platform and 150 bp paired-end reads were generated.

### 2.3. mRNA Identification and Differential Expression Analysis

HISAT2 [29] was used to obtain location information of reference genomes or genes, as well as unique sequence feature information of sequencing samples. Based on the comparison software Bowtie2 [30], HISAT2 recognizes splicing junctions between exons. DEseq2 [31] estimated the expression levels of all transcripts. After generating the final transcriptome, Bowtie2 and eXpress [32] were used to estimate the expression levels of all transcripts. The expression level of each transcript in each sample was calculated according to the FRKM (Fragments Per kb Per Million Reads) [33] method. Two standard FoldChange and FDRs (adjusted *p*-values) were selected for DEmRNAs analysis of the same transcript in two samples. DEmRNAs with |log2(fold change)| > 1 and *p* ≤ 0.05 were extracted.

GO enrichment analysis was performed on DEmRNAs to describe their function combined with GO annotation results from eggNOG-mapper (http://eggnog-mapper.embl.de/, accessed on 28 January 2023) with hypergeometric distribution test for the significance, the results were visualized using the WEGO website (https://wego.genomics.cn/, accessed on 28 January 2023).

### 2.4. Identification and Phylogenetic Tree Analysis of DcNAC Family in RJ 

To identify the NAC family members, we downloaded all the genome data and annotation information of RJ from NCBI (https://www.ncbi.nlm.nih.gov/ accessed on 28 January 2023) and searched for the NAC domain from the pfam database with a hidden Markov model (PF02365) (http://www.sanger.ac.uk/Software/Pfam/ accessed on 28 January 2023). The potential NAC family genes in RJ were searched for using HMMER3.0 (e-value < 1 × 10^−5^). All obtained sequences were validated and filtered through the SMART database (http://smart.embl-heidelberg.de/, accessed on 28 January 2023). After removing genes that do not have NAC domains and duplicate transcripts of the same gene, a total of 89 DcNAC family genes were found in *D. catenatum.*

MUCLE software (version 5) was used to perform multiple sequence alignment on 89 DcNAC proteins. The phylogenetic tree was constructed using MEGA7 using the neighbor-joining method with a bootstrap test (1000 replicates). ITOL was used to modify and visualize the phylogenetic trees (https://itol.embl.de/, accessed on 29 January 2023).

### 2.5. Expression Pattern Analysis of 32 DEDcNACs in Various Tissues

The expression data of different tissues, including root (SRX2938667), stem (SRR4431600), leaf (SRR4431601), sepal (SRR4431597), labellum (SRR4431602), pollinia (SRR5722145) and gynostemium (SRR4431596), were downloaded from National Center for Biotechnology Information website (https://www.ncbi.nlm.nih.gov/ accessed on 28 June 2023). The expression patterns of 32 DE*DcNACs* were visualized using TBtools (https://pubmed.ncbi.nlm.nih.gov/32585190/, accessed on 8 June 2023).

### 2.6. Methods of Stress Treatment, RNA Extraction, and Quantitative/Real-Time-PCR (qRT-PCR) Analysis

For ABA treatment, we used 100 μM ABA to spray on the surface of RJ seedlings. For NaCl treatment, we soaked the seedlings of RJ in 300 mM NaCl solution. After four hours of treatment, the mixture of leaves and stems treated with ABA and NaCl among RJ seedlings cultured under normal conditions was collected for RNA extraction. 

Total RNA was extracted using Total RNA Extract Reagent (Coolaber, Beijing, China, Cat# RE600), and cDNA was synthesized using the HiScript Q RT SuperMix for qPCR (+gDNA wiper) (Vazyme, Nanjing, China, Cat# R123-01) according to the manufacturer’s instructions. qRT-PCR was performed to investigate the expression levels of 32 DE*DcNACs* under ABA and NaCl treatment. The qRT-PCR reactions were performed on the CFX Real-time PCR system (BIORAD, Berkeley, CA, USA, Cat# 1855201) using ChamQ SYBR qPCR Master Mix (Vazyme, Nanjing, China, Cat# Q311-02/03). The qRT-PCR primers of these 32 genes are listed in Table S3.

### 2.7. ROS Staining

The RJ seedlings were treated with drought, ABA, and NaCl treatment, respectively. The seedlings were soaked in 100 μM ABA and 300 mM NaCl for 4 h, and a 50 mL centrifuge filled with absorbent silica gel for 8 h.

To detect accumulation of H_2_O_2_ and O_2_^−^, DAB and NBT staining were performed using 50 mM NaAc-HAc containing 1 mg/mL DAB (pH = 3.8), 25 mM HEPES buffer (pH = 7.6) containing 1 mg/mL NBT, respectively. CK and treated seedlings were placed in DAB and NBT stain for ten minutes of vacuum shocks, and then incubated at room temperature in the dark for 2 h. The samples were decolorized with 95% ethanol until all chlorophyll was removed. The samples were observed and photographed using a stereo optical microscope, and the “ImageJ” software (version 2.0) was used to quantify the stained area.

### 2.8. Identification and Analysis of lncRNAs

A comprehensive comparison and analysis of spliced transcripts were conducted against reference transcripts to identify known encode transcripts or novel transcripts that align with known loci. The cuffcompare software (version 2.0) (https://github.com/gpertea/CuffCompare accessed on 28 June 2023) was used to compare and analyze the merged transcripts with the reference transcript. Then, the transcripts that completely match or are similar to the known lncRNA were screened out. Simultaneously, the specific location type of the remaining transcripts was determined. We reserved transcripts with the words ‘i’, ‘u’, ‘x’, and ‘o’ as candidate lncRNA transcripts. Next, the candidate lncRNA transcripts were screened according to a length greater than 200 bp and the number of exons greater than or equal to 2. The transcripts screened in the second step were predicted and analyzed using CPC (Coding Potential Calculator), CNCI (Coding-Non-Coding index), Pfam, and PLEK [34,35,36,37] to screen out the transcripts with potential coding function. 

### 2.9. Identification and Analysis of circRNAs

CIRI (CircRNA Identifier) software (version 1.0) was used to predict circRNAs from scratch [38]. CIRI software (version 1.0) uses BWA software (version 0.7.17.) to compare with the reference gene sequence to generate a Sequence Alignment/Map (SAM) file, and identifies circRNAs by scanning SAM files twice. During the first scan of SAM files, CIRI uses paired chiastic clipping (PCC), paired-end mapping (PEM), and GT-AG splicing signals to detect junction reads. For additional junction read detection and further filtering of false positives, the second scan of SAM files was used. For double-ended reads, the CIRI algorithm will consider a pair of reads, one of which can be mapped to circRNA, and the other also needs to be mapped to the range of circRNA. For splicing signal (GT-AG), CIRI will also consider other weak splicing information. The algorithm extracted the exon boundary position from the GTF/GFF file, and filtered the false positive with the known boundary.

Two standards were used to compare and analyze whether there is differential expression of the same circRNA in two samples of CircSeq data. The default condition for filtering the difference is *p* < 0.05 and FoldChange > 2.

### 2.10. Identification and Analysis of miRNAs

The primers and vector sequences were removed from the original Fastq file data and quality testing and length screening were performed on the sequencing fragment bases. High-quality and reliable sequencing fragments were selected, and small RNA fragments with a length of 18–30 nt were finally screened out. Then, the clean reads sequence was BLAST searched against the Rfam database (version 14.9, https://rfam.org/ accessed on 8 July 2023) to remove small RNAs such as tRNA, snRNA, rRNA, and cRNA. Subsequently, the filtered sequence was compared with all mature miRNA sequences in miRbase (version 21.0, http://www.mirbase.org/ accessed on 8 July 2023), and the known miRNA precursor and mature body sequences were compared and annotated using Bowtie software. Mireap software (version 0.20.) (http://sourceforge.net/projects/mireap accessed on 8 July 2023) was used to perform a novel miRNA prediction on sequences that are not annotated. The miRNAs screened from different samples were statistically analyzed. The number of reads was tested for significance of difference using NB (negative binomial distribution test). The expression of miRNAs was estimated using the basemean value, and the miRNAs with *p* value < 0.05 and multiple changes occurring more than twice were screened.

### 2.11. ceRNA Network Construction and Analysis

The regulatory relationship of miRNA–target (miRNA–mRNA, miRNA–lncRNA, and/or miRNA–circRNA) was predicted using psRNATarget (http://plantgrn.noble.org/psRNATarget/home accessed on 8 July 2023). The expression value was used to predict the miRNA–target, mRNA–lncRNA, and mRNA–circRNA relationship pairs, and screen out the miRNA–target regulatory relationships with negative correlations in the expression. The *p* value of the correlation coefficient of expression value is <0.05, and the Pearson correlation coefficient is >0.8 or <−0.8. The expression value and the prediction result of the target gene were integrated to obtain the ceRNA relationship pair. The Cytoscape software (version 3.6.1) was used to visualize the ceRNA network [39].

## 3. Results

### 3.1. Evaluation of Tolerance to Drought Stress in D. catenatum

**Figure 1:**
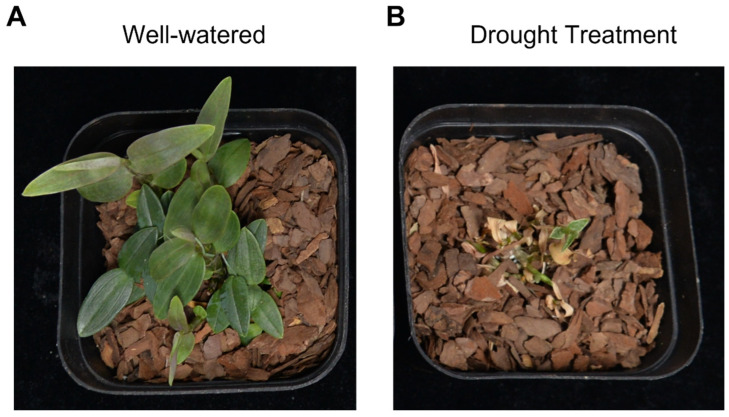
Well-watered plant (**A**) and drought-treated plant (**B**) of species *D. catenatum* (RJ) under 25 °C, 16 h of illumination, and 8 h of darkness conditions.

In this study, the *D. catenatum* variety ‘Honggan ruanjiao’ (RJ) was selected for the drought experiment to assess its drought response. Since *D. catenatum* is a highly drought-tolerant plant, we conducted a two-month extreme drought treatment and monitored the phenotypic changes. The results indicated that compared to the plants grown under well-watered conditions (Figure 1A), the plants that were not watered still exhibited green stems, albeit with less vigorous growth (Figure 1B). Consequently, these drought-stressed materials were employed for a comprehensive transcriptome analysis.

### 3.2. The Global Responses of mRNA to Drought Stress

**Figure 2:**
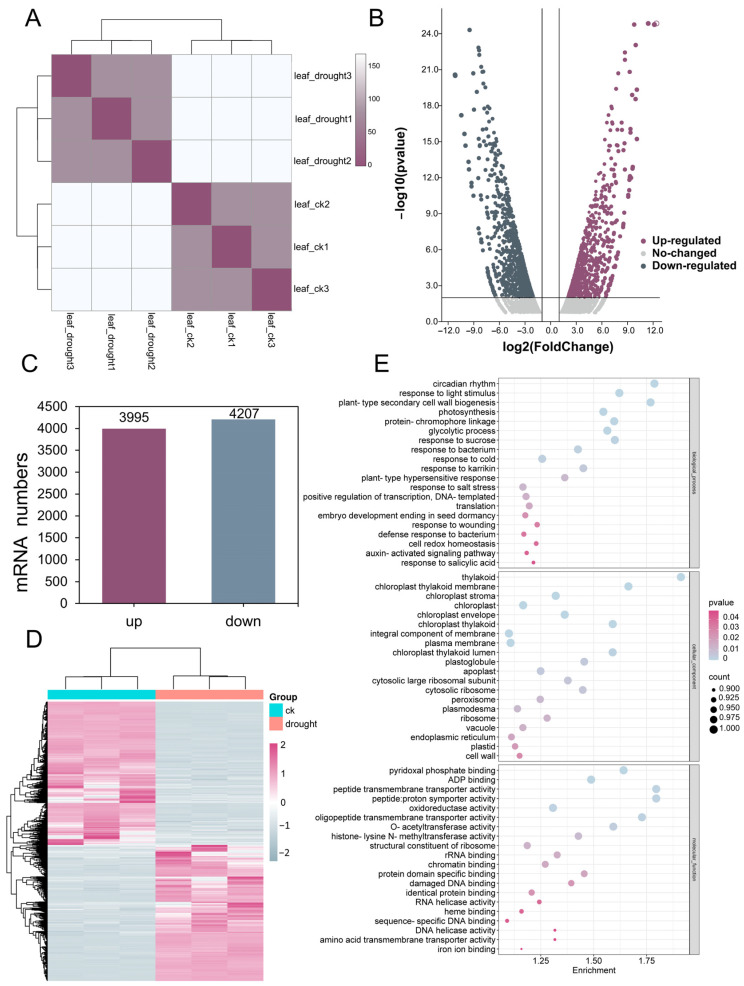
Identification and analysis of differentially expressed mRNAs (DEmRNAs) in response to drought treatment. (**A**) Sample clustering analysis of three biological replicates of CK and drought-treated samples; (**B**) volcano plot displaying the identification of DEmRNAs in CK and drought treatment samples with significance plotted against fold change; (**C**) statistical analysis presenting the number of up- and down-regulated DEmRNAs; (**D**) heat map showing the expression pattern of DEmRNAs in three replicates of both CK and drought-treated samples; (**E**) Gene Ontology (GO) classifications of the DEmRNAs.

Transcriptome sequencing was performed on three replicates of both the control group and the drought treatment group. A total of 16,911,055 and 18,347,376 clean reads were obtained from the CK and drought-treated samples, respectively, using *D. catenatum* as the reference genome. Sample clustering analysis was conducted on three biological replicates of the CK and drought-treated samples, respectively (Figure 2A). The two clusters of the CK and drought-treated samples can be significantly distinguished. The mRNA abundance of each transcript was assessed using FPKM and baseMean. To identify differentially expressed mRNAs (DEmRNAs), a threshold of |log2(fold change)| > 2 and *p* ≤ 0.05 was applied (Figure 2B). A total of 8202 DEmRNAs were identified, including 3995 up-regulated and 4207 down-regulated transcripts (Figure 2C, Table S1). The expression patterns of the DEmRNAs in the CK and drought-treated samples were visualized through a heatmap (Figure 2D), demonstrating the DEmRNAs in a group of CK and drought-treated samples were clustered separately, while the three biological replicates were clustered together. The specific up-regulated and down-regulated mRNAs clustered in Figure 2D can be found in Table S1. It is worth noting that various transcription factor families have been found in these DEmRNAs, such as NAC, WAKY, MYB, bHLH, and ERF.

Gene Ontology (GO) and Kyoto Encyclopedia of Genes and Genome (KEGG) enrichment analyses were performed to gain insights into the function of the DEmRNAs. The GO enrichment results (Figure 2E) revealed that the DEmRNAs are predominantly involved in various biological processes (BPs) such as circadian rhythm, plant-type secondary cell wall biogenesis, photosynthesis, protein–chromophore linkage, the glycolytic process, and the response to light and sucrose. In terms of the cellular components (CCs), the DEmRNAs were highly enriched in thylakoid, chloroplast thylakoid membrane, chloroplast thylakoid, and chloroplast thylakoid lumen, indicating their involvement in a series of molecular mechanisms within the chloroplast in response to drought stress. Furthermore, the DEmRNAs displayed significant enrichment in molecular function (MF) terms such as peptide transmembrane transporter activity, peptide proton symporter activity, oligopeptide transmembrane transporter activity, pyridoxal phosphate binding, and *O*-acetyltransferase activity. This suggested that the DEmRNAs may regulate membrane channels to respond to drought stress. These findings provided valuable insights into the potential regulatory mechanisms of the identified DEmRNAs in response to drought stress in RJ. Therefore, further investigation of these regulatory mechanisms could lead to a deeper understanding of the drought response pathways and molecular adaptations in RJ.

### 3.3. Identification and Analysis of NAC Transcription Factor Family of DEmRNAs in RJ 

Transcription factors have emerged as critical regulatory factors governing various aspects of plant growth, development, and response to abiotic stress. Several studies have focused on exploring transcription factor families in *D. catenatum*, including AP2/ERF, WOX, TCP, MYB, LBD, WRKY, HD-ZIP, MADS-box, and bHLH [40,41,42,43,44,45,46,47]. However, the characterization of the NAC transcription factor family in RJ remains unexplored.

**Figure 3:**
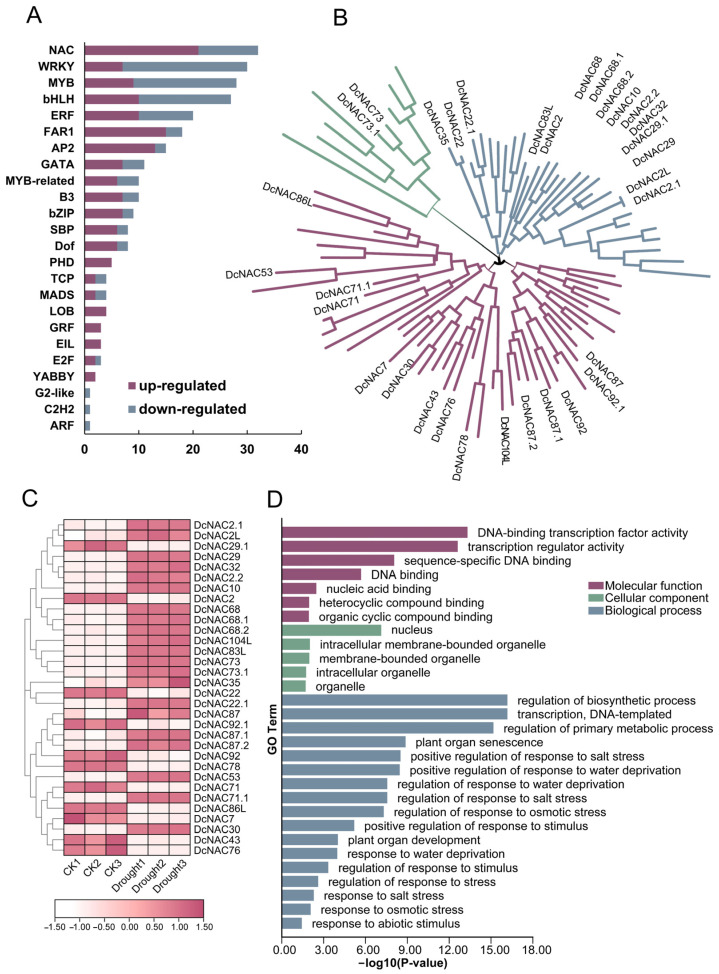
Analysis of DEmRNAs and NAC family in RJ. (**A**) Column diagram displaying the number of different types of transcription factors in DEmRNAs; (**B**) phylogenetic tree of the NAC family in *D. catenatum* was constructed using neighbor-joining method with 1000 bootstrap replications. The three branches were color-coded, and the DE*DcNAC* genes were labeled in black. (**C**) Heatmap of the expression patterns of differentially expressed *DcNAC* (DE*DcNAC*) gene in the control group and drought group under three biological replicates. The outer circle of the heat map represents the DE*DcNAC* evolutionary tree. (**D**) Gene Ontology (GO) classification.

Our analysis of DEmRNAs in RJ under control and drought treatment revealed that NAC transcription factors account for the most up-regulated numbers (21) of differentially expressed transcription factors (Figure 3A). This finding highlighted that the NAC transcription factor family plays crucial roles in the drought resistance of *D. catenatum*. Moreover, we analyzed the NAC family in RJ with the HMM model and identified a total of 89 NAC family members (Table S2). Subsequently, a phylogenetic tree was constructed based on these NAC transcription factors, which were categorized into three branches (Figure 3B). Among the 89 NAC family members, 32 were expressed differentially between CK and treatment (Figure 3C). Notably, the phylogenetic analysis showed that these 32 NAC transcription factors were distributed across all three branches. 

To explore the mechanism of the NAC family in the drought resistance of *D. catenatum*, GO analysis was conducted (Figure 3D). The results showed that the NAC family is deeply involved in a variety of biological processes (BPs) to respond to abiotic stresses, especially those caused by drought or water shortage including “positive regulation of response to salt stress”, “positive regulation of response to water deprivation”, “positive regulation of response to stimulus”, and “response to osmotic stresses”. The enrichment of these GO terms of biological processes directly revealed the role of NAC transcription factors in plant drought response signaling pathways. For molecular function (MF), a total of seven terms were found and all of these were consistent with the characteristics of transcription factors. Five terms were found under cellular component (CC), which included nucleus, intracellular membrane-bound organelle, membrane-bound organelle, intracellular organelle, and organelle. Collectively, our results indicated that the 32 differentially expressed NAC transcription factors were involved in drought stress responses in RJ.

### 3.4. Tissue Expression Patterns of Differentially Expressed NAC Transcription Factors in RJ 

**Figure 4:**
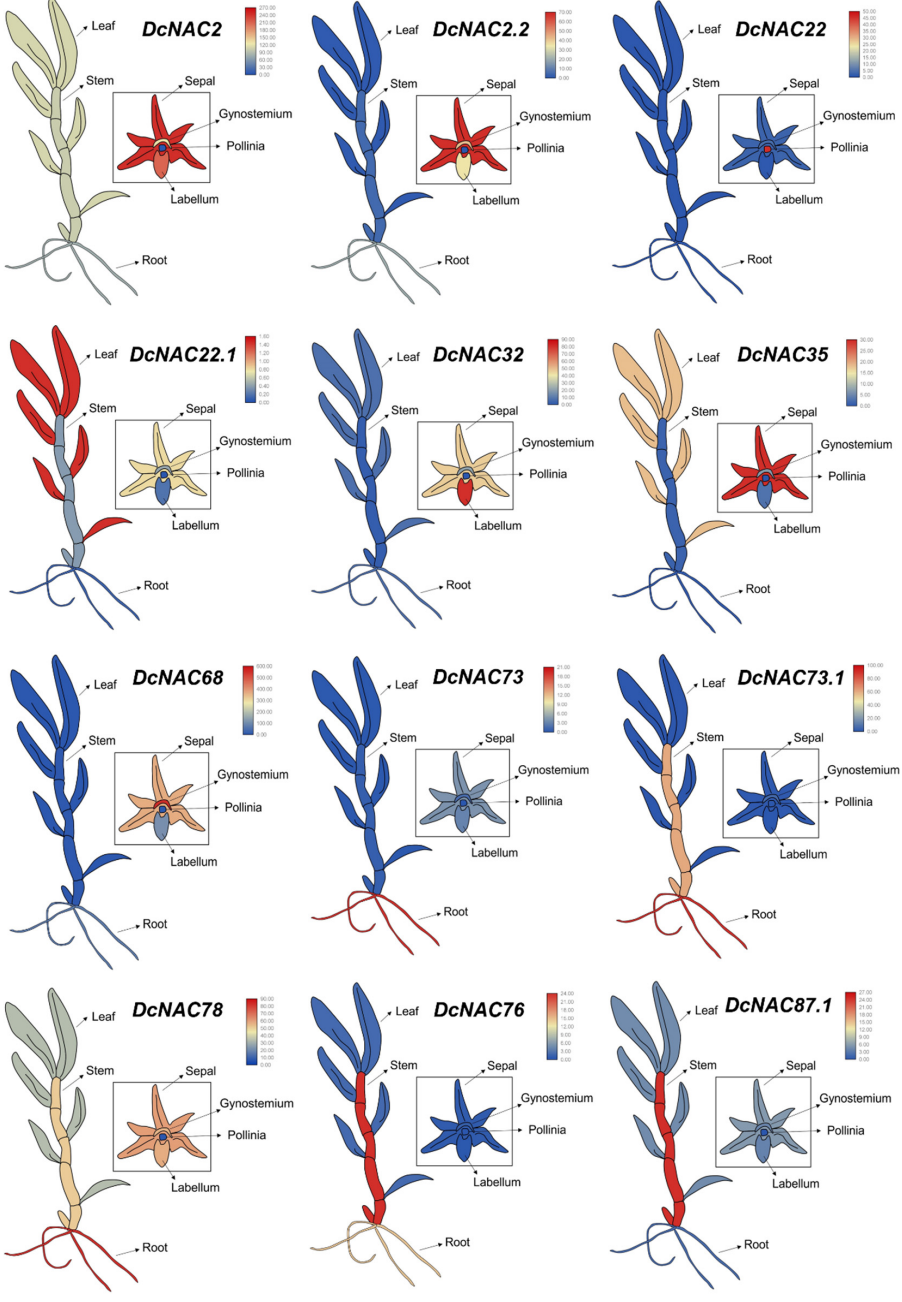
The expression patterns of DE*DcNAC* genes in different tissues of RJ.

To comprehensively analyze the expression patterns of DE*DcNAC* transcription factors, NAC expression profiles were represented using cartoon graphics based on transcriptome data from different tissues (roots, stem, leaves, and floral organ) in RJ (Figure 4 and Figure S1). Figure 4 depicted 12 distinct expression patterns of the 12 DE*DcNACs*. Among the 32 analyzed DE*DcNACs*, 20 were found to be predominantly expressed in the floral organs including sepal, gynostemium, pollinia, and labellum. Notably, *DcNAC68.1*, *DcNAC30*, *DcNAC68,* and *DcNAC71* exhibited specific expression in the gynostemium. Eleven genes tended to be expressed in the root, with only two genes (*DcNAC76* and *DcNAC87.1*) being expressed mostly in the stem, and one gene (*DcNAC22.1*) being mainly expressed in the leaves (Figure S1). Importantly, *DcNAC2.1* and *DcNAC7* were found to be expressed in both the root and floral organs, indicating their potential involvement in the drought stress response. These findings demonstrated that the DE*DcNACs* were responsive to drought stress in various tissues of RJ.

### 3.5. Response of DEDcNACs under ABA and NaCl Treatment

Since osmotic stress can be induced by both drought stress and high salinity stress, we subjected RJ plants to ABA and NaCl treatments to investigate the responses of the 32 DE*DcNACs* to abiotic stresses. The qRT-PCR primers for these 32 genes are listed in Table S3.

**Figure 5:**
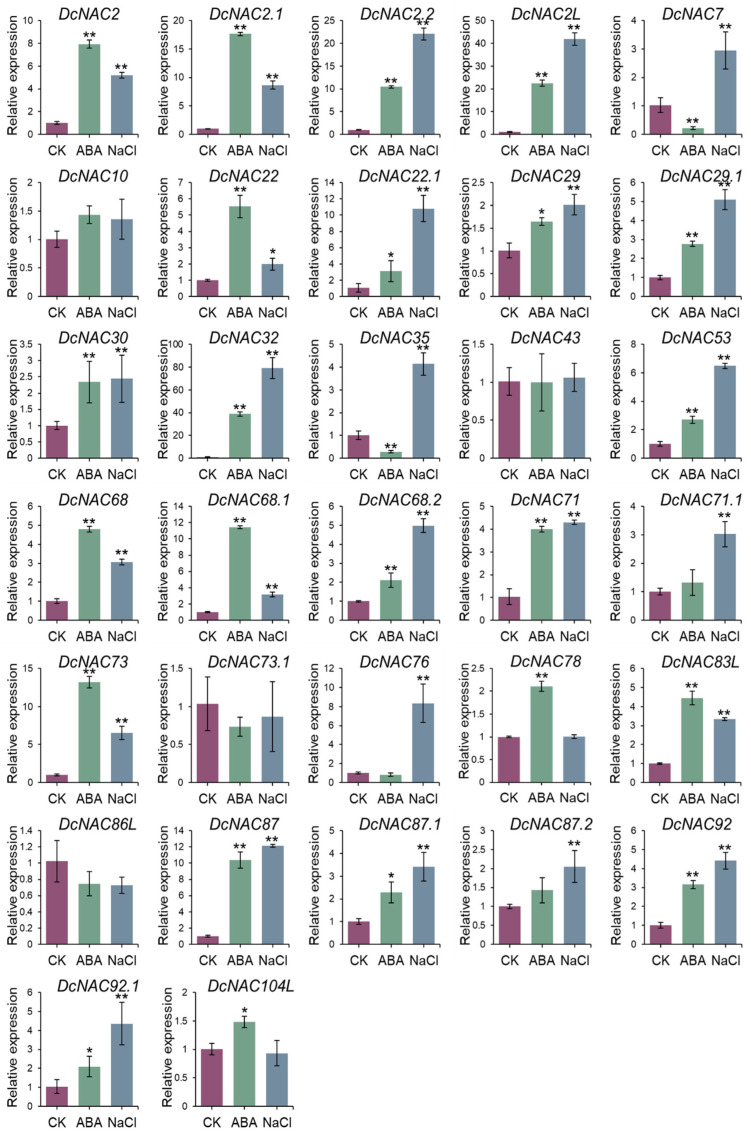
Expression patterns of 32 DE*DcNAC* genes under ABA and NaCl treatment. The purple, green, and blue columns represented CK, ABA treatment, and NaCl treatment, respectively. *DcACTIN2* was used as internal control. Values were presented as means ± SD (*n* = 3). (* *p* < 0.05, ** *p* < 0.01, Student’s *t*-test).

As shown in Figure 5, the expression of *DcNAC2*, *DcNAC2.1*, *DcNAC2L*, *DcNAC22*, *DcNAC32*, *DcNAC68.1*, and *DcNAC73* were significantly increased under the ABA treatment, surpassing the expression levels in the control (CK) by more than eight-fold. Meanwhile, the expression levels of *DcNAC78* and *DcNAC104L* also increased under ABA treatment. Twelve genes showed increasing expression levels under both the ABA and NaCl treatments, but the expression levels of *DcNAC2.2*, *DcNAC2L*, *DcNAC22.1*, *DcNAC32,* and *DcNAC53* significantly increased only under the NaCl treatment. Interestingly, *DcNAC7* and *DcNAC35* showed decreased expression under the ABA treatment, while they significantly increased under the NaCl treatment. *DcNAC30*, *DcNAC71,* and *DcNAC87* exhibit equivalent increases in expression under both treatments. There was no significant change in the expression levels of *DcNAC43* and *DcNAC73.1* under both the ABA and NaCl treatments. These findings indicated that these 32 DE*DcNACs* were involved in the response to osmotic stresses in RJ through multiple pathways.

### 3.6. ROS Levels in RJ under Different Treatments

**Figure 6:**
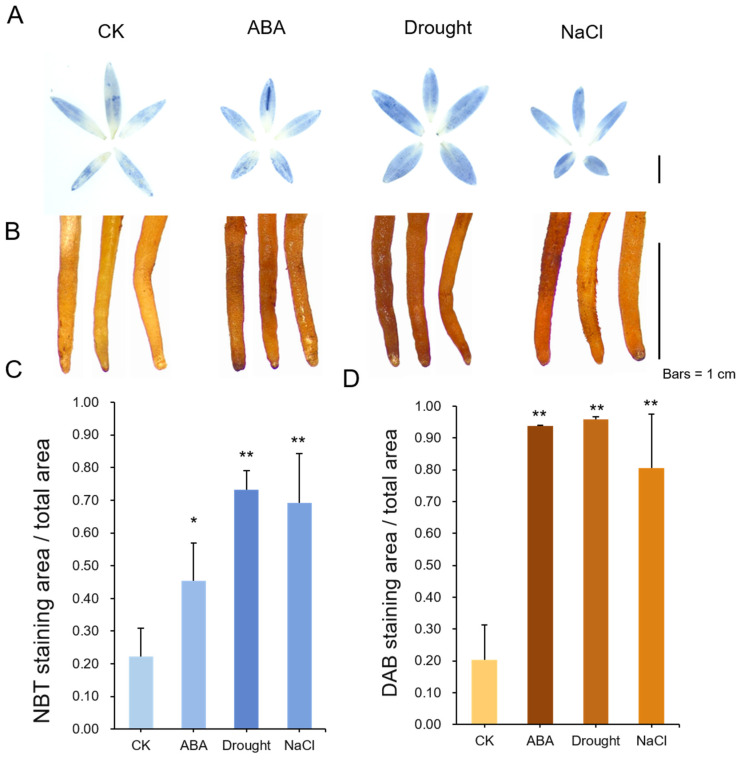
ROS levels in RJ under ABA, drought, and NaCl treatments. (**A**) NBT staining for superoxide; (**B**) DAB staining for H_2_O_2_; (**C**) relative NBT staining intensities; (**D**) relative DAB staining intensities, (Student’s *t*-test; * *p* < 0.05, ** *p* < 0.01, *n* = 10); Bars = 1 cm.

Since drought stress leads to an increase in ROS (reactive oxygen species) levels, the accumulation of H_2_O_2_ and O_2_^−^ under drought, ABA, and NaCl treatments in RJ were examined using DAB and NBT staining. Figure 6A showed the NBT staining of RJ leaves under different treatments. The staining of the drought-treated seedlings was stronger than those of the ABA-treated and NaCl-treated seedlings, indicating that O_2_^−^ accumulated most under the drought treatment. In addition, we performed DAB staining to detect the accumulation of H_2_O_2_. The results also showed the staining was strongest under the drought treatment (Figure 6B). The quantification of the NBT and DAB staining is shown in Figure 6C,D. It is worth mentioning that the DAB staining intensity of the seedlings treated with ABA was stronger than that of the seedlings treated with NaCl, but the opposite results were observed in the NBT staining, indicating that ROS levels are increased through different pathways in RJ.

### 3.7. The Transcriptome-Wide Responses of lncRNA to Drought Stress 

**Figure 7:**
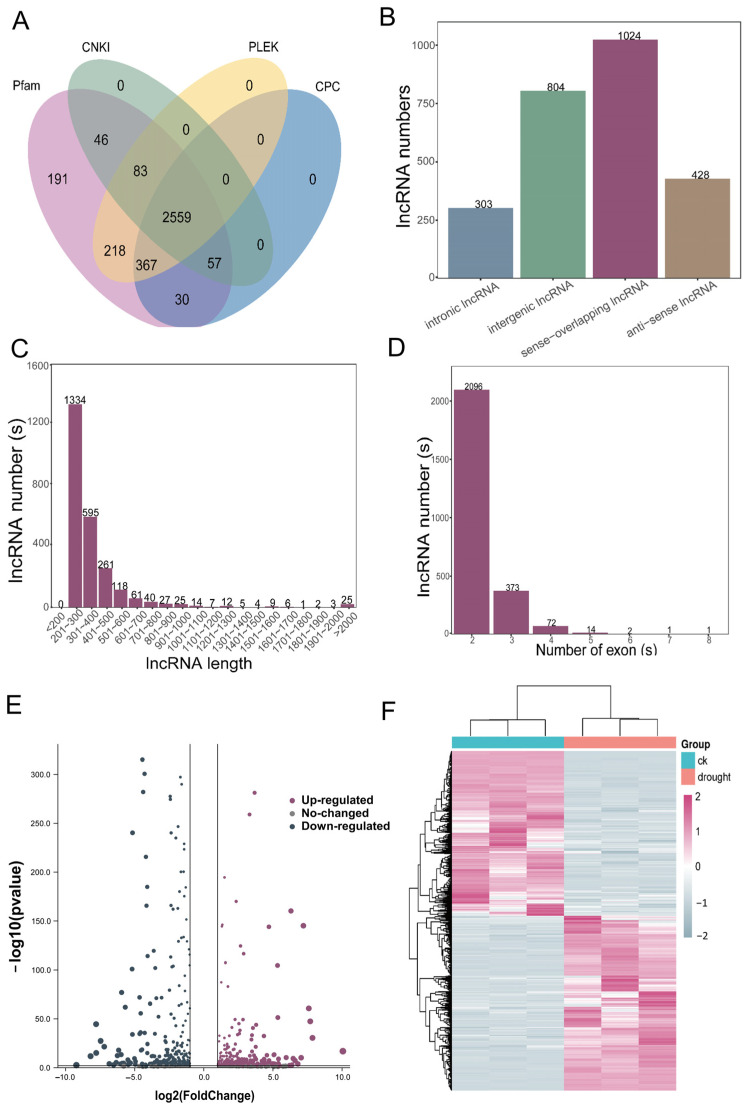
Identification and analysis of DElncRNAs under drought stress. (**A**) Venn diagram of DElncRNAs in CK and drought treatment samples; (**B**) the quantitative distribution analysis of various types of DElncRNAs; (**C**) the length distribution of DElncRNAs; (**D**) the distribution of exon numbers in DElncRNAs; (**E**) volcano plot of log2 FoldChange and *p*-values (padj) of DElncRNAs in CK and drought treatment samples; (**F**) heat map of DElncRNAs in CK and drought treatment samples.

A comprehensive analysis of the long non-coding RNAs (lncRNAs) was conducted, using CPC, CNCI, PLEK, and Pfam analysis, resulting in the identification of 2559 lncRNAs (Figure 7A, Table S4). These lncRNAs were categorized into different classes, including 303 intronic lncRNAs, 804 intergenic lncRNAs, 1024 sense-overlapping lncRNAs, and 428 anti-sense lncRNAs (Figure 7B). The majority of the lncRNAs were found to be between 200 and 300 bp in length, and most of the lncRNAs were less than 1000 bp (Figure 7C). The number of lncRNA exons was analyzed. Figure 7D showed that more than 80% of the lncRNAs exhibited two exons, while a smaller proportion had three (373 lncRNAs), four (72 lncRNAs), or five exons (2 lncRNAs). Only two lncRNAs contained seven and eight exons, respectively.

The differential transcripts were further analyzed through a volcano plot analysis (Figure 7E). Among all these DElncRNAs, 460 were up-regulated and 437 were down-regulated. Two criteria were utilized to identify differential expression transcripts: FoldChange and FDR (adjusted *p*-value). The default screening condition was |log2(fold change)| > 2 and *p* ≤ 0.05. A cluster analysis performed on the DElncRNAs is shown in Figure 7F and all these clustered lncRNAs can be found in Table S1. Similar to the DEmRNAs, the results showed that three replicates of each treatment clustered together, while the CK and drought treatment samples were grouped separately, indicating good reproducibility among the CK and drought-treated transcripts.

### 3.8. The Global Expression Patterns of circRNA to Drought Stress

**Figure 8:**
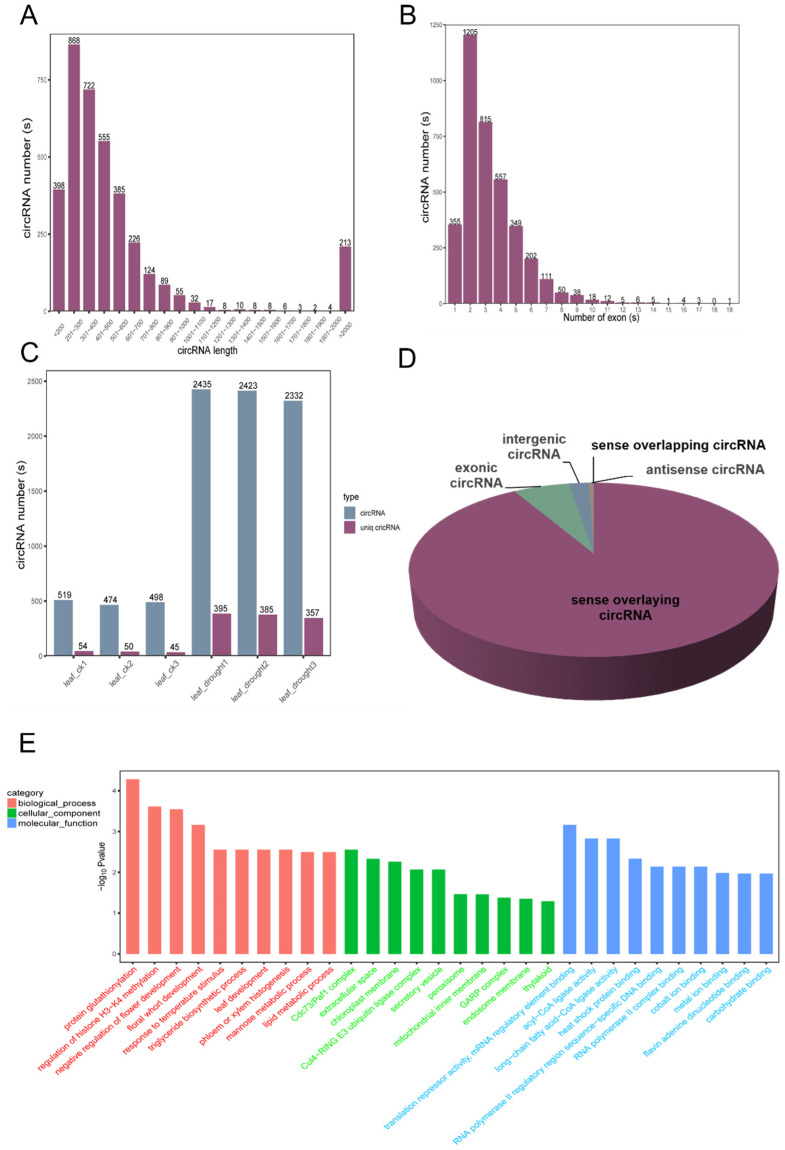
Identification and analysis of DEcircRNAs under drought stress. (**A**) Length distribution of DEcircRNAs; (**B**) distribution of exon numbers in DEcircRNAs; (**C**) distribution of predicted circRNAs in each sample; (**D**) quantitative distribution of different types of DEcircRNAs; (**E**) Gene Ontology (GO) classification of DEcircRNAs.

Similar to lncRNAs, the majority of the circRNAs (868) were found to be between 200–300 bp, with only 5.7% (213) of the lncRNAs measuring more than 2000 bp (Figure 8A). Through analyzing the number of exons in the circRNA sequences, it was observed that most of the circRNAs contained fewer than seven exons. Notably, the circRNAs with two exons were the most abundant, comprising a maximum of 1205 occurrences, while the circRNAs with the highest number of exons contained up to 19 (Figure 8B). In total, 3737 circRNAs were identified in the CK and drought samples (Table S5), with 45–54 unique to the CK samples and 357–395 unique to the drought-treated samples (Figure 8C). The results indicated both the total number of circRNAs and unique circRNAs were higher in the drought-treated samples compared to the control samples. 

Based on the genomic location, the circRNAs can be divided into five categories (Figure 8D). The sense overlapping circRNAs consist of the smallest proportion, accounting for only 0.03%. The exonic circRNAs make up 5.86%, the intergenic circRNAs account for 2.14%, the antisense circRNAs represent 0.43%, and the highest content of sense overlapping circRNAs was 91.54%.

Similarly, FoldChange and FDR (|log2(fold change)| > 2 and *p* ≤ 0.05) were selected as the criteria to screen the differentially expressed circRNAs (DEcircRNAs). A total of 326 DEcircRNAs were identified, with 226 up-regulated and 100 down-regulated. After obtaining the DEcircRNAs, GO enrichment analysis was performed in order to explore how these DEcircRNAs function under drought stress (Figure 8E). The GO terms corresponding to more than two circRNAs in three classifications were screened, and the top 30 terms were selected for GO enrichment analysis. The most enriched terms in the BPs included protein glutathionylation, the regulation of histone H3-H4 methylation, histone methylation, and the glutathione metabolic process. For the CCs, the Cdc73/Paf1 complex was highlighted, while RNA polymerase complex binding featured prominently in Molecular Function (MF). Furthermore, the KEGG enrichment analysis revealed the involvement of the “Metabolic pathways” and “Biosynthesis of secondary metabolites” pathways. Collectively, these results indicated that the circRNAs likely play crucial roles in drought stress through the pathways associated with these enriched terms.

### 3.9. The Integrated Small RNA Response to Drought Stress in D. catenatum

**Figure 9:**
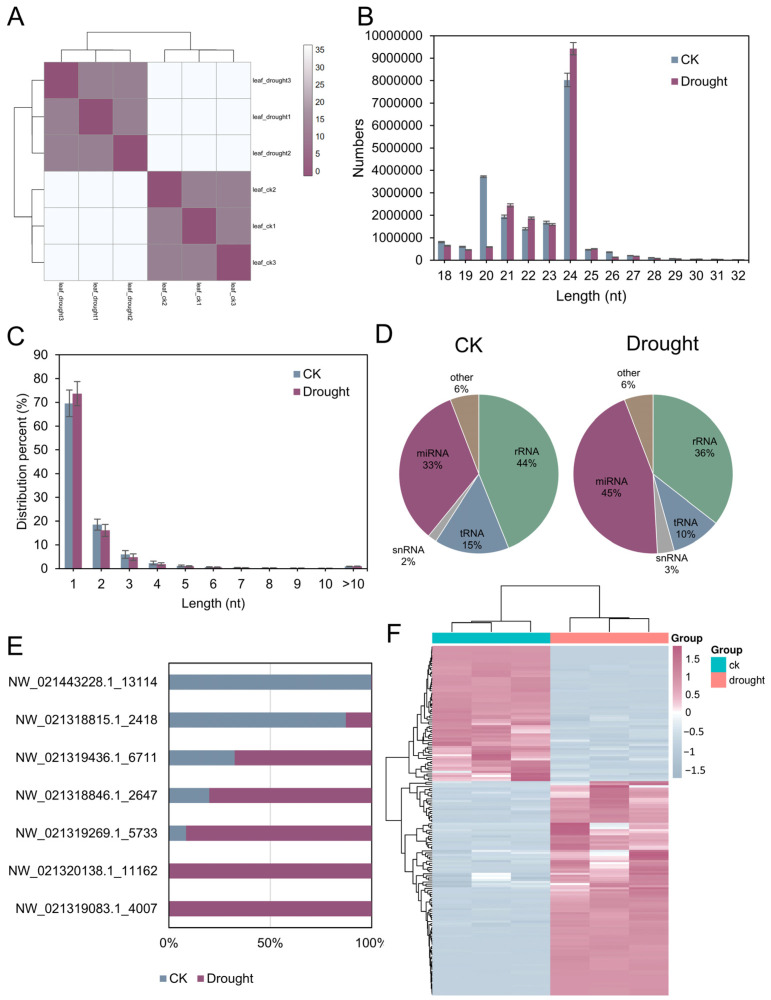
Identification and analysis of miRNAs under CK condition and drought stress. (**A**) Sample clustering analysis of three biological replicates of CK and drought-treated samples; (**B**) length distribution of sRNAs; (**C**) distribution of predicted miRNAs in each sample; (**D**) quantitative distribution of different types of sRNAs; (**E**) top five expressed novel miRNAs in each treatment; (**F**) heat map of the expression patterns of DEmiRNAs in three replicates of both CK and drought-treated samples.

All the small RNAs (sRNAs) obtained from this study were represented in Table S6. A sample clustering analysis of the miRNAs was conducted on three biological replicates of the CK and drought samples, respectively. The two clusters of the CK and drought-treated samples can be significantly separated (Figure 9A). The length distribution of the sRNAs typically fell within the length range of 18–30 nt. Upon analyzing the transcriptome of six samples, we observed that all these sRNAs were predominantly distributed at a length of 24 nt (Figure 9B). The varying numbers of reads (unique reads) in each sample are illustrated in Figure 9C. the miRNAs containing a single copy number accounted for the highest proportion in the control samples and drought-treated samples, accounting for 69.57% and 73.69%, respectively. Comparing the different types of small RNAs between the drought treatment and CK samples, the miRNA percentages increased from 33% to 45% and snRNA increased from 2% to 3%, while rRNA and tRNA decreased by 8% and 5%, respectively (Figure 9D). We summarized the top five novel RNAs with high expression levels from the control group and drought group, respectively; among them, three RNAs were duplicated. Subsequently, we obtained the proportion of the remaining seven novel RNAs in the expression levels of the two groups of samples (Figure 9E). Interestingly, NW_021319083.1_4007 and NW_021320138.1_11162 were exclusive to the drought samples, while NW_021443228.1_13114 and NW_021318815.1_2418 accounted for a significant proportion in the control group, indicating that these novel miRNAs are likely potential regulatory molecules in drought stress. The cluster analysis of the six transcripts and differentially expressed miRNAs (DEmiRNAs) revealed that the three duplicated drought samples and three duplicated control samples were, respectively, clustered together. Moreover, the DEmiRNAs consistently exhibited distinct changes in both treatments (Figure 9F).

### 3.10. CeRNA Regulatory Network in Response to Drought Stress

To gain insight into the intricate interplay among the different RNA molecules in RJ during the drought treatment, a competitive endogenous RNA (ceRNA) regulatory network was constructed using DEmiRNAs, DEmRNAs, DElncRNAs, and DEcircRNAs, based on the ceRNA theory (Figure S2, Table S7). The ceRNA hypothesis has revealed that ceRNAs regulate the expression of transcripts by competing with microRNA response elements (MREs) that are shared with mRNA. Consequently, the MREs presented on each transcript collectively form the basis for ceRNA interaction network regulation [48]. For RNA transcription, no matter whether they encode a protein or not, they can compete with each other to combine with microRNA and regulate each other, thus forming a huge ceRNA network (ceRNETs).

The ceRNA networks of RJ yielded 206 nodes, with 136 up-regulated and 152 down-regulated miRNAs identified. The ceRNA relationship pairs were obtained by integrating the expression value and target gene prediction results. Further screening was carried out based on the following criteria: common miRNAs were different; ceRNA *p* value < 0.05; common miRNA of ceRNA ≥ 10. In total, 100 DEmRNAs, 32 DElncRNAs, and 25 DEcircRNAs were predicted as targets of 74 miRNAs (Figure S2).

**Figure 10:**
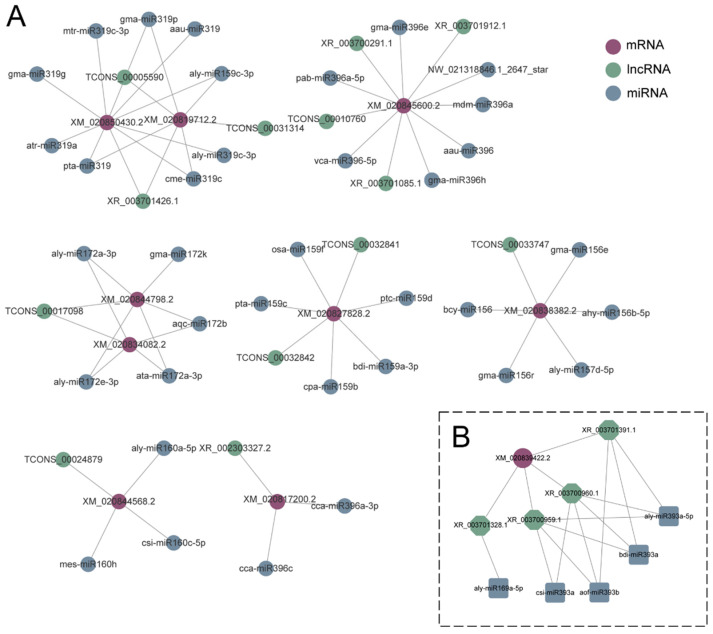
Mini-ceRNA network constructed with 7 known drought-related DEmRNAs, DElncRNAs, DEmiRNAs in RJ (**A**), and mini-ceRNA network constructed with *DcNAC87* (**B**). Colored circles represented the DEmRNAs (purple), DEmiRNAs (blue), and DElncRNAs (green).

To simplify the overwhelming amount of information in the ceRNA network, we created a mini-ceRNA network focusing on the mRNAs associated with drought stress (Figure 10), including mRNAs related to cell walls: XM_020817200.2 (regulated by XR_002303327.2 and miR396), XM_020827828.2 (regulated by TCONS_00032841 and miR159), XM_020850430.2 (regulated by XR_003701426.1 and miR319); auxin related protein: XM_020844798.2 (regulated by TCONS_00017098 and miR172), XM_020844568.2 (regulated by TCONS_00024879 and miR160); genes related to ABA degradation: XM_020845600.2 (regulated by TCONS_00010760, XR_003700291.1, XR_003701085.1, XR_003701912.1, miR396 and NW_021318846.1_2647_star); serine/threonine-protein kinase: XM_020838382.2 (regulated by TCONS_00033747, miR156 and miR157); strigolactone esterase: (regulated by TCONS_00017098 and miR172); and transcription factor: XM_020819712.2 (regulated by XR_003701426.1, TCONS_00005590, TCONS_00031314, miR159 and miR319). It is worth noting that we found *DcNAC87* to be predicted in the ceRNA network and may be regulated by miRNA169 and miRNA393, as well as lncRNAs like XR_003701328.1, XR_003700959.1, XR_003700960.1, and XR_003701391.1. Among them, XR_003701328.1, XR_003700959.1, XR_003700960.1, and XR_003701391.1 were involved in the regulation of 29, 16, 16, and 20 mRNAs in the ceRNA network, respectively. Next, we conducted tissue expression analysis on these four lncRNAs and found that the expression patterns of XR_003701328.1 and XR_003701391.1 were highly similar to those of *DcNAC87* (Figure S3). Therefore, we reasonably speculate that XR_003701328.1 and XR_003701391.1 are the main lncRNAs that are involved in the regulation of *DcNAC87* under drought stress.

## 4. Discussion

Drought stress is a serious challenge for plant growth [49]. It is crucial to cultivate drought-resistant varieties, excavate drought-resistant genes, and reveal drought-resistant mechanisms. Although many drought-related genes and non-coding RNAs in plants have been analyzed, the study of the transcriptome in *D. catenatum,* which has a strong drought tolerance, has not been conducted yet. Therefore, the sequencing of its transcriptome will help to excavate the drought-tolerance-related protein-coding RNA and non-coding RNA, as well as analyze the role of various RNA molecules in the ceRNA network. 

ABA is one of the important hormones in regulating abiotic stress in plants. We found that 46 DEmRNAs were discovered to be up-regulated in the ABA-activated signaling pathway, such as XM_028694637.1 (MA16_Dca026261), a serine/threonine-protein kinase SAPK3. OsSAPK3, a member of the sucrose non-fermenting 1-related kinases 2 (SnRK2s) family, could improve the drought tolerance and increase the yield of rice [50]. The experiments on the ABA induction of salt-tolerant varieties and salt-sensitive rice showed that *SAPK7* (MA16_Dca026261) and *WRKY24* could be induced by ABA [51]. The expression of serine/threonine-protein kinase (*SAPK7*) and transcription factor *WRKY24* in *D. catenatum* were also up-regulated under drought stress, indicating that they may also cope with drought by responding to ABA signals. 

It is widely known that transcription factors regulate plant growth, development, as well as stress defense responses through specific interactions with various cis-acting elements of genes. NAC is one of the largest transcription factor families in plants and participates in numerous processes within the plant like plant development and biological and abiotic stress responses. There have been reports on the drought response of the NAC family in Arabidopsis [52], rice [53], soybean [54], pearl millet [55], peanut [56], maize [57], and other species. In the analysis of the tissue expression patterns of the 32 DE*DcNACs* identified in this study, we found that most DE*DcNACs* were expressed in the floral organ, and secondly in the root. It has been reported that hormone biosynthesis and transportation, as well as abiotic stresses, such as heat, cold, and drought, usually affect the development and fertility of the floral organ. Former studies on the NAC family ANAC019 in Arabidopsis have found that *ANAC019* can not only be expressed in floral organs [58], but also participates in ABA- and JA-mediated signaling pathways [59,60], and then regulate genes involved in flower development in early drought response to maintain reproductive development [61]. *TaRNAC1* in wheat is a constitutive transcription factor mainly expressed in roots. The overexpression of *TaRNAC1* can improve drought tolerance and increase grain yield [62]. In drought-tolerant rice lines, the expression of the *NAC78* gene is specifically induced [63]. These studies all demonstrate the important role of NAC transcription factors in different tissues under drought stress. 

These 32 DE*DcNACs* are not only expressed in different tissues, but also participate in drought through different modes of osmotic stress. Based on the long-term chronic drought treatment transcriptome data (Figure 3C) and the expression patterns under the acute ABA and NaCl treatments of 32 DE*DcNACs* (Figure 5), we have some interesting findings. The expression levels of *DCNAC2*, *DCNAC22*, and *DCNAC78* increased under the ABA treatment, indicating that they participate in drought stress through ABA-dependent pathways, and are downstream, physiologically activated genes of the ABA signaling pathway. However, the expression levels are significantly lower under long-term chronic drought stress than those of the well-watered condition, so they may play different roles under acute and chronic drought stresses. The expression levels of *DCNAC7* and *DCNAC76* decreased under both drought and ABA conditions, but increased under NaCl treatment. Therefore, they are likely to balance osmotic stress by negatively regulating drought stress and positively regulating salt stress. However, the expression level of *DCNAC86L* decreased under all these stresses, so it may be a negative regulatory factor in osmotic stress pathways. Previous studies have shown that NAC can regulate drought stress through ABA-dependent, ABA-independent, and high osmotic stress pathways [64,65,66]. These studies have indicated the important role of NAC transcription factors in drought stress, and our identification of DE*DcNACs* will further provide new insights into the drought resistance mechanism in *D. catenatum*.

With the proposal of the ceRNA hypothesis, some studies have shown that different RNA small molecules also play important roles under drought stress. Since miRNAs play crucial roles in the ceRNA network, we identified several key mRNAs that participated in the ceRNA network of RJ under drought treatment. XM_020845600.2 is the abscisic acid 8′-hydroxylase CYP707A2-like gene and is related to ABA degradation. In Arabidopsis, HISTONE DEACETYLASE 9 (HDA9) and ABI4 can form a repressive complex that inhibits the expression of CYP707A1 and CYP707A2 in response to drought stress [67]. Under severe drought, with the concentration of ABA increases, the ABA catabolism-related gene CYP707A2 is down-regulated [68]. XM_020823235.2 encodes a γ-aminobutyric acid (GABA) transporter. GABA, a non-protein amino acid, plays a significant role in the response of plants to drought, salt, and heavy metal stresses. It has been found to alleviate oxidative damage by regulating antioxidants [69]. As microRNAs are a central factor in the ceRNA network, exploring the role of them will help us screen more drought-related mRNAs and lncRNAs based on the constructed ceRNA network. According to the DEmiRNAs identified in this study, miR159 [16], miR169 [70], and miR393 [71] have been reported to be involved in the ABA-dependent pathway, while several other miRNAs, such as miR156 [72], miR160 [73], and miR167 [74] can regulate the plant drought response through ABA-independent pathways. In this study, we have identified that *NAC87* (XM_020839422.2) from the DE*DcNACs* can be regulated by miR169, miR393, XR_003701328.1, XR_003700959.1, XR_003700960.1, and XR_003701391.1 in the ceRNA network. Since miR169 can affect the sensitivity of plants to ABA, and miR393 can affect genes related to stomatal development, we have reason to believe that *NAC87* may be involved in the drought regulation of *D. catenatum* through ABA-dependent pathways. Further research on drought-related miRNAs and target genes will promote the understanding of the molecular mechanisms by which plants respond to drought stress.

## 5. Conclusions

In this study, we systematically analyzed the transcriptome of *D. catenatum* under CK and drought treatment using whole-transcriptome RNA-seq data. From the transcriptome data, the mRNA, lncRNA, circRNA data were analyzed and a ceRNA network consisting of differentially expressed mRNAs, miRNAs, and lncRNAs was constructed. This further revealed the critical roles and potential interaction relationship of these DERNAs in tolerance to drought stress. In addition, we innovatively identified a prominent transcription factor family NAC in *D. catenatum*. The tissue expression profiles, qRT-PCR results, and GO terms indicate that NAC transcription factors actively respond to drought stress in *D. catenatum*. Overall, these results are of great significance for characterizing drought resistance genes and lay the foundation for studying how different types of RNA respond to the drought stress of *D. catenatum*.

## Data Availability

Data is contained within the article and supplementary material.

## Supplementary Materials

The following supporting information can be downloaded at: https://www.mdpi.com/article/10.3390/antiox13010094/s1. Figure S1: Tissue expression patterns of the rest 20 differentially expressed *DcNACs* in different tissues of RJ; Figure S2: Drought stress response ceRNA network constructed with all DEmRNAs, DElncRNAs and DEmiRNAs; Figure S3: Tissue expression patterns of *DcNAC87*, XR_003701328.1, XR_003700959.1, XR_003700960.1 and XR_003701391.1 in leaf, stem, and root tissues of RJ; Table S1: mRNA list; Table S2: Basic information of *DcNAC* Gene family; Table S3: RT-qPCR primer list of DE*DcNAC*; Table S4: lncRNA list; Table S5: circRNA list; Table S6: small RNA list; Table S7: ceRNA data.
