# Supplementary material for: Whole-Transcriptome Sequencing Reveals the Global Molecular Responses and NAC Transcription Factors Involved in Drought Stress in Dendrobium catenatum
Source: Antioxidants (Basel). 2024 Jan 12;13(1):94. doi: 10.3390/antiox13010094 (PMC10812421; doi:10.3390/antiox13010094)
Supplement: Supplementary file 1 [file antioxidants-13-00094-s001.zip › Supplementary Fig S1~3.pdf]

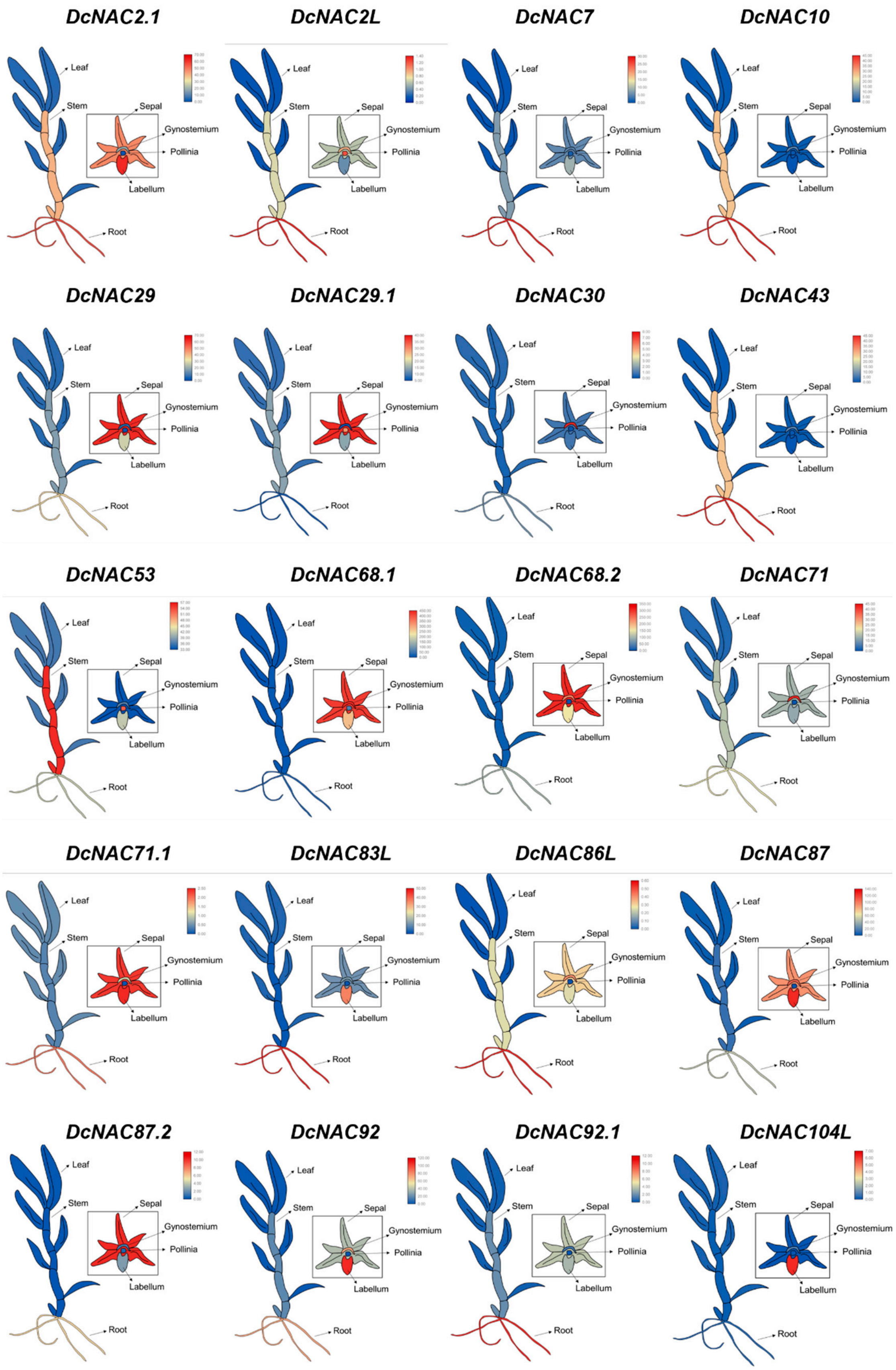

Figure S1. Tissue expression patterns of the rest 20 differentially expressed *DcNAC* in different tissues of RJ

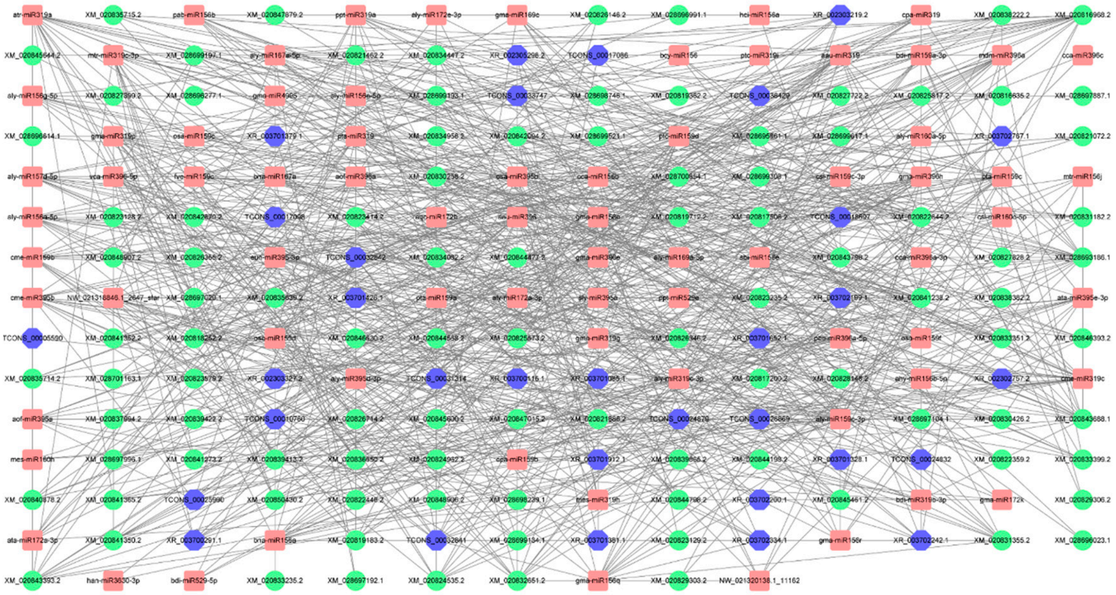

Figure S2. Drought stress response ceRNA network constructed with all DEmRNAs, DElncRNAs and DEmiRNAs. The color represents DEmRNAs (green color), DEmiRNAs (red color), and DElncRNAs (dark blue color)

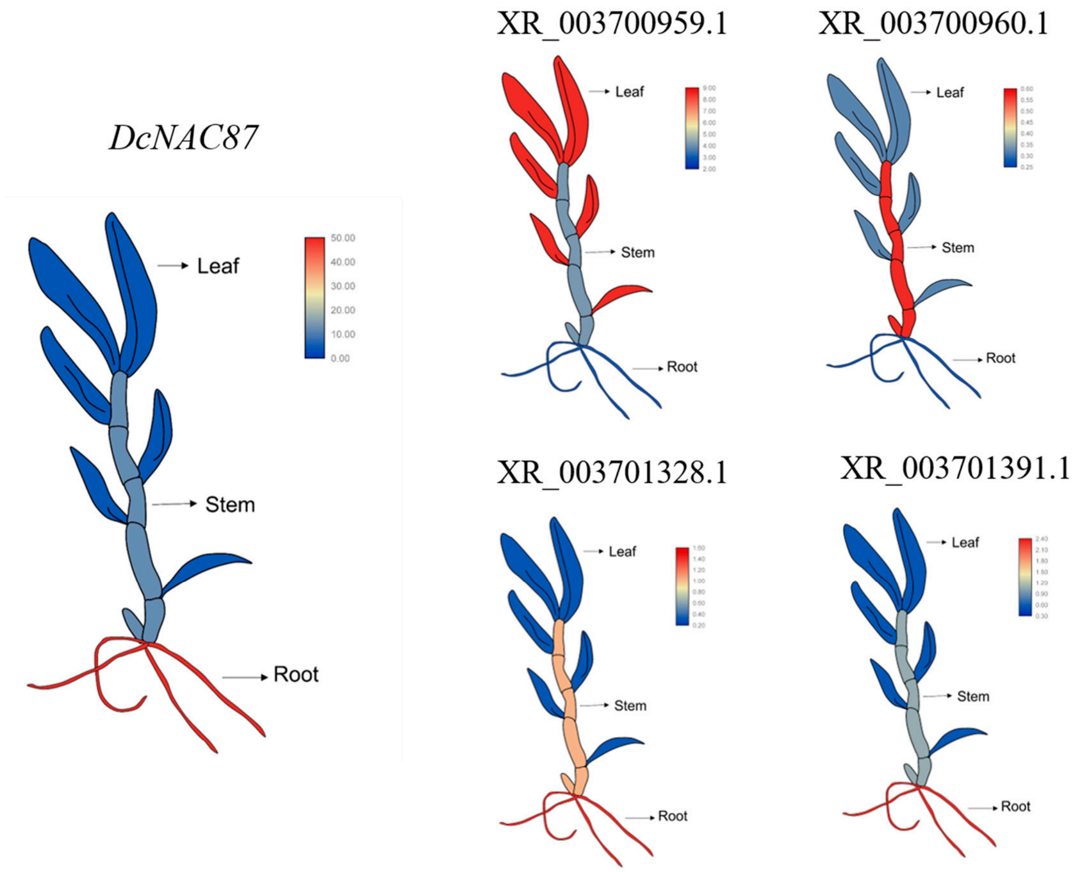

Figure S3. Tissue expression patterns of XR_003701328.1, XR_003700959.1, XR_003700960.1 and XR_003701391.1. in different tissues of RJ.
